# Spiritual well-being and perceived stress in primary health care nursing professionals

**DOI:** 10.1590/0034-7167-2024-0193

**Published:** 2025-06-20

**Authors:** Fabiana Gonçalves Seki Gava, Ruth Natalia Teresa Turrini

**Affiliations:** I Universidade de São Paulo. São Paulo, São Paulo, Brazil

**Keywords:** Stress, Psychological, Spirituality, Nursing, Primary Health Care, Cross-Sectional Studies., Estrés Psicológico, Espiritualidad, Enfermería, Atención Primaria de Salud, Estudios Transversales.

## Abstract

**Objective:**

To examine the relationship between spiritual well-being and perceived stress levels in primary health care nursing professionals.

**Methods:**

This descriptive and correlational study was conducted with 87 nursing professionals. Data were collected using the Perceived Stress Scale and the Spiritual Well-being Scale. The data were then analyzed using Spearman’s correlation, simple linear regression, and the Kruskal-Wallis test.

**Results:**

A significant difference was observed in perceived stress levels across high, moderate, and low categories of spiritual well-being, with a medium effect size. Spiritual well-being showed a moderate negative correlation with perceived stress levels.

**Conclusion:**

Higher levels of spiritual well-being are associated with lower levels of perceived stress, suggesting that spirituality may be an effective coping tool for managing stress among primary health care nursing professionals.

## RESUMEN

**Objetivo:**

Verificar la relación entre el bienestar espiritual y los niveles de estrés percibido en profesionales de enfermería de atención primaria de salud.

**Métodos:**

Estudio descriptivo y correlacional realizado con 87 profesionales de enfermería. Para la recolección de datos se utilizaron la Escala de Estrés Percibido y la Escala de Bienestar Espiritual. Los datos fueron sometidos a análisis de correlación de Spearman, regresión lineal simple y prueba de Kruskal-Wallis.

**Resultados:**

Se observó una diferencia significativa en los niveles de estrés percibido entre las categorías de bienestar espiritual alto, moderado y bajo, con un tamaño de efecto medio. El bienestar espiritual mostró una correlación negativa moderada con los niveles de estrés percibido.

**Conclusión:**

Niveles más altos de bienestar espiritual están asociados con niveles más bajos de estrés percibido, lo que sugiere que la espiritualidad puede ser una herramienta eficaz para enfrentar el estrés en los profesionales de enfermería de atención primaria.

## INTRODUCTION

Perceived stress can be defined as a situation that an individual perceives as significant, challenging, or threatening, where the demands exceed their resources to cope^(1)^. Stress should not be understood as something exclusively negative to be avoided. The human body is adapted to handle stress in small doses, as it stimulates action, enabling individuals to focus their attention and make optimal decisions under certain circumstances. However, when stress intensifies in both intensity and duration, it becomes harmful. Prolonged high levels of stress interfere with mental balance and cognitive and functional abilities. They are also associated with adverse health behaviors, insulin resistance, diabetes, metabolic syndrome, hypertension, osteoporosis, central obesity, dyslipidemia, and depression^(2,3)^.

Primary health care (PHC) is the main entry point for individuals into the public health system and is responsible for addressing up to 90% of the population’s health issues^(4,5)^. The extensive scope of PHC, combined with the specific demands of this model, can contribute to the development of mental health issues among professionals in this field, particularly nursing staff. The shortage of human resources results in work overload and insufficient time to perform necessary tasks. This, combined with conflicts with team members and patients, role ambiguity, lack of support from supervisors, and feelings of powerlessness in the face of patient suffering, may contribute to increased stress levels among PHC nursing staff^(6)^. Work-related stress places these professionals at greater risk of developing physical and mental health problems, impacting their personal, family, and professional lives. Thus, there is a need to adopt strategies to promote health and resilience among this population^(7,8)^, with the cultivation of spirituality standing out.

The literature does not present a consensus on the definition of spirituality. However, most studies describe spirituality as a personal search for meaning and purpose in life, for answers to existential questions, and for understanding one’s relationship with the sacred or transcendent, which may or may not be related to religion^(9)^. Spirituality is a dimension present in all human beings, whereas religion is associated with specific beliefs and formal practices. Spirituality can be seen as a guiding element, capable of providing a sense of direction in the pursuit of improved physical, socio-emotional, intellectual, occupational, and environmental health^(10)^.

Spiritual well-being is considered a dimension of health and can be defined as a positive emotional state with constructive behaviors and uplifting thoughts in one’s relationships with oneself, with others, and with a dimension beyond the tangible. This state fosters a sense of identity, fulfillment, contentment, affection, consideration, optimism, inner serenity, balance, purpose, and meaning in life^(10)^.

When individuals live with a spirituality-based perspective, they exhibit greater tolerance for physical and psychological stress and demonstrate improved coping abilities when faced with serious illness and isolation, which can even contribute to successful aging^(11)^. Spirituality positively influences resilience and hope and empowers health professionals to handle experiences of pain, suffering, and death^(11)^. Furthermore, spiritual well-being is associated with increased job satisfaction and enhanced professional performance^(12,13)^. This state improves adaptive skills and mental function, having a beneficial impact on nursing care. A significant positive correlation exists between nursing care and spiritual well-being^(13)^.

Despite the growing number of studies on the importance of spiritual care, most research focuses on spiritual care for patients rather than for nursing professionals. Few studies have examined the relationship between spiritual well-being and stress levels in this population^(8)^. Two studies conducted in the United States, one with pediatric nurses and another with nursing students, indicated a weak inverse correlation between levels of spiritual well-being and perceived stress^(12,14)^. No national studies on this topic were found.

## OBJECTIVE

To examine the relationship between spiritual well-being and perceived stress levels in PHC nursing professionals.

## METHODS

### Ethical aspects

The study was conducted in accordance with national and international ethical guidelines and was approved by the Research Ethics Committees of the School of Nursing and the School of Public Health at the University of São Paulo. Informed consent was obtained electronically from all participants.

### Study design, period, and location

This is a descriptive and correlational study, designed according to the STROBE (Strengthening the Reporting of Observational Studies in Epidemiology) recommendations for cross-sectional studies^(15)^.

Data were collected from 32 out of 40 Basic Health Units (UBS in Portuguese) in the city of Osasco, SP, Brazil, and from the Geraldo de Paula Souza Health School Center (CSEGPS in Portuguese) in São Paulo, SP, Brazil, between October 2021 and June 2022.

### Sample, inclusion and exclusion criteria

The population consisted of nursing assistants, technicians, and nurses. The non-probabilistic sample included 87 nursing professionals working in the UBS who voluntarily participated in the study. The inclusion criteria were identifying as stressed and having worked at the institution for at least six months. Professionals who were on vacation or leave during the data collection period were excluded.

### Study protocol

For participants from the Osasco UBS units, the invitation to participate in the study was sent by the nursing coordinators of the PHC department via WhatsApp® in the work group. The message included an explanatory video and a link to the electronic form for indicating participation interest, which contained contact information and questions regarding eligibility criteria. At the CSEGPS, the invitation was made during a team meeting, in which one of the researchers explained the study objectives and distributed leaflets with a QR code linking to the electronic form for indicating participation interest.

Eligible participants received the electronic data collection form via WhatsApp®, which included the Informed Consent Form (ICF), the biosociodemographic questionnaire, the Perceived Stress Scale (PSS), and the Spiritual Well-being Scale (SWBS).

By clicking the link to access the data collection form, participants were automatically directed to the ICF page, which contained all information about the study and the researchers’ contact details. After reading the ICF, participants selected one of three options: A) “I agree to voluntarily participate in the study”; B) “I do not agree to participate in the study”; C) “I have questions about the study and would like clarifications before agreeing to participate.” The instrument could only be accessed if the participant chose option A.

The biosociodemographic instrument included information related to professional category, gender, marital status, age, and number of children.

The PSS measures the degree to which individuals perceive situations in their lives as unpredictable, uncontrollable, and overwhelming^(16)^. The instrument was validated for Brazilian Portuguese, with internal consistency measured by Cronbach’s alpha at 0.82 in the validation study and 0.91 in the present study. The scale consists of 14 items on a Likert scale, with responses ranging from 0 (never) to 4 (always), with scores reversed for negative items. The total score ranges from zero to 56 points and can be categorized into five scoring intervals: <18 = low stress; 19-24 = normal stress; 25-29 = moderate stress; 30-35 = high stress; and >35 = very high stress^(17,18)^.

The SWBS includes 20 items divided into two subscales with ten items each: Religious Well-being (RWB), which measures the individual’s satisfaction with God, and Existential Well-being (EWB), which assesses whether the individual has a sense of life purpose, independent of religion^(19)^. The scale was validated for Brazilian Portuguese, with internal consistency measured by Cronbach’s alpha at 0.92 for the overall scale, 0.92 for RWB, and 0.85 for EWB^(20)^. In the present study, internal consistency values were 0.92 for the overall scale, 0.82 for RWB, and 0.94 for EWB. Responses are recorded on a Likert scale ranging from 1 (strongly disagree) to 6 (strongly agree), with scores reversed for negative items. The total score ranges from 20 to 120, and the categorization suggested by the authors is as follows: for SWBS, 20-40 = low spiritual well-being, 41-99 = moderate spiritual well-being, 100-120 = high spiritual well-being; for the RWB subscale, 20-40 = unsatisfactory relationship with God or low RWB, 21-49 = moderate sense of religious well-being or moderate RWB, 50-60 = positive view of the relationship with God or high RWB; and for the EWB subscale, 10-20 = low life satisfaction and possible lack of clarity of life purpose or low EWB, 21-49 = moderate level of life satisfaction and purpose or moderate EWB, 50-60 = high level of life satisfaction and a clear sense of purpose or high EWB^(21)^.

### Data analysis and statistics

To characterize the sample, descriptive measures of absolute and relative frequencies, as well as measures of central tendency and variability, were used.

The correlation between the PSS and the SWBS, including its subscales RWB and EWB, was assessed using Spearman’s correlation. To evaluate the strength of the correlations, the following parameters were adopted: < 0.3 = weak correlation, 0.3 to < 0.7 = moderate correlation, and ≥ 0.7 = strong correlation. Independent variables that showed a significant correlation with the outcome were included in a simple linear regression model.

To compare PSS levels according to the SWBS categories and its subscales, the Kruskal-Wallis test was used, followed by Dunn’s post hoc test for category comparison. Effect size was measured using rank eta squared (η^2^), with the following classifications: 0.02 = small, 0.13 = medium, and 0.26 = large^(22)^.

Simple bivariate linear regression analyses were conducted between sociodemographic variables and SWBS, with those having p < 0.2 included in a multiple linear regression model along with SWBS.

Data were stored in a Microsoft Excel® spreadsheet and analyzed using JAMOVI software, version 2.4.2. The level of significance was set at 5%.

## RESULTS

A total of 113 nursing professionals completed the electronic form expressing interest in participating. There was an attrition of 26 participants: two did not meet the inclusion criteria, and 24 withdrew before completing the data collection instrument, resulting in a final sample of 87 participants.

Table 1 presents the biosociodemographic characteristics of the study participants. The median PSS score aligns with the high stress classification, with a prevalence of 52.8% (n=46) of participants reporting high or very high stress levels. The median SWBS score classifies participants within the high spiritual well-being range (50.6%, n=44). Regarding the subscales, the medians classify participants as having a positive view of their relationship with God (high RWB - 64.4%, n=56) and a moderate level of life satisfaction and sense of purpose (moderate EWB - 67.8%, n=59).

**Table 1:** Biosociodemographic characteristics of participants. Osasco and São Paulo, São Paulo, Brazil, 2022

Qualitative Variables (N=87)	n (%)	
Sex		
Female	80 (92.0)	
Male	7 (8.0)	
Marital status		
Single	20 (23.0)	
Married or in a stable union	50 (57.5)	
Divorced	14 (16.1)	
Widowed	3 (3.4)	
Professional category		
Technical level	61 (70.1)	
Nurse	26 (29.9)	
**Quantitative Variables (n=87)**	**Mean (SD)**	**Median (1stQ; 3rdQ)**
Age	44.7 (9.3)	44.0 (38.0; 50.5)
Number of children	1.6 (1.2)	1.0 (1.0; 2.0)
PSS	31.1 (8.2)	30.0 (25.5; 37.5)
RWB	50.1 (12.3)	55.0 (43.0; 60.0)
EWB	42.2 (9.6)	43.0 (36.5; 50.0)
SWBS	92.2 (20.0)	100.0 (82.5; 106.0)

*Note: EWB = Existential Well-being; SWBS = Spiritual Well-being; RWB = Religious Well-being; SD = Standard Deviation; PSS = Perceived Stress; 1stQ = First Quartile; 3rdQ = Third Quartile.*

**Figure 1:**
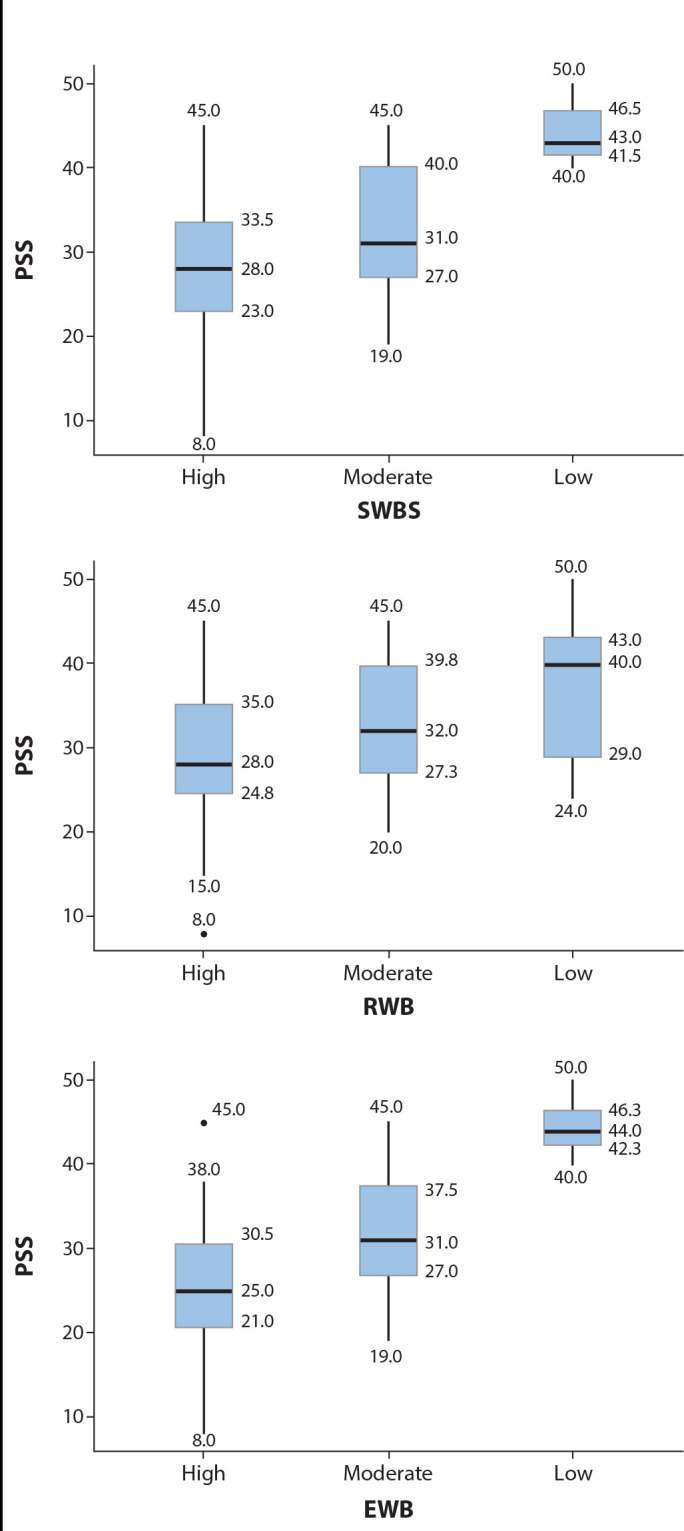
Distribution of median PSS Scores by categories of Spiritual Well-being and its subscales. Osasco and São Paulo, São Paulo, Brazil, 2022

*Note: SWBS = Spiritual Well-being; RWB = Religious Well-being; EWB = Existential Well-being; PSS = Perceived Stress.*

When observing the distribution of PSS scores according to the SWBS categories and its subscales, it is evident that the higher the spiritual well-being, the lower the levels of perceived stress (Figure 1). Among participants with high SWBS, RWB, and EWB, the median PSS score was classified as moderate. For participants with moderate SWBS, RWB, and EWB, the median PSS score was classified as high. Finally, among participants with low SWBS, RWB, and EWB, the median PSS score was classified as very high.

The Kruskal-Wallis test was conducted to compare the median PSS scores across the categories of SWBS, EWB, and RWB. A significant difference was found between categories for SWBS (p = 0.002) and EWB (p < 0.001), but not for RWB (p = 0.06). Dunn’s post hoc test for multiple comparisons was then conducted to verify differences in PSS between the categories of spiritual well-being for the variables with statistically significant differences. For SWBS, the test showed significant differences between the high and moderate categories (p = 0.015), high and low categories (p = 0.004), and moderate and low categories (p = 0.05), with a medium effect size (η^2^ = 0.119). The same pattern was observed for EWB, with differences between high and moderate categories (p = 0.004), high and low categories (p < 0.001), and moderate and low categories (p = 0.011), also with a medium effect size (η^2^ = 0.180).

When analyzing the association between spiritual well-being and stress, a moderate and significant negative correlation was observed between SWBS and PSS levels, as well as between EWB and PSS levels. No significant correlation was found between RWB and PSS, as shown in Table 2.

**Table 2:** Correlation between PSS, SWBS, and its Subscales. Osasco and São Paulo, São Paulo, Brazil, 2022

Variables	PSS	SWBS	EWB	RWB
PSS	1.000	--	--	--
SWBS	-0.367^*^	1.000	--	--
EWB	-0.519^*^	0.897^*^	1.000	--
RWB	-0.122	0.842^*^	0.568^*^	1.000

*Note: p < 0.001. SWBS = Spiritual Well-being; EWB = Existential Well-being; RWB = Religious Well-being; PSS = Perceived Stress.*

SWBS and EWB were included in a simple linear regression analysis to verify their relationship with the PSS variable. RWB was not included, as it did not show a significant correlation with the outcome variable. In this model, both EWB (p < 0.001; r^2^ = 0.304) and SWBS (p < 0.001; r^2^ = 0.154) were found to be negatively associated with PSS.

Sociodemographic variables were not associated with PSS levels: age (p = 0.697), number of children (p = 0.959), marital status (p = 0.146), and professional category (p = 0.405). Marital status, due to having a p-value < 0.2, was included in a multiple regression model along with SWBS, but the analysis did not show significant results for marital status categories (married-single, p = 0.361; divorced-single, p = 0.509; widowed-single, p = 0.714). In this model, only SWBS was associated with PSS levels (p = 0.001).

## DISCUSSION

The majority of study participants were women, mid-level professionals living with a partner, with an average age over 40 years and few children. Nursing is a profession historically practiced by women. Although the number of men in nursing has increased over the past 30 years, they still represent only 14.1% of the workforce in this field^(23)^. Most participants were in the third phase of their professional lives, the maturity phase, which includes individuals between 36 and 50 years old^(24)^. These results are similar to those of a study conducted with nursing professionals in Brazil during the COVID-19 pandemic^(23)^, but differ from the general nursing profile in the country, where 61.7% of professionals are under 40 years old^(24)^. The data on professional category and marital status also align with the nursing profile in Brazil, where 77% of professionals are at the technical level and 48.7% are married, in a stable union, or in a consensual contract^(24)^.

Nursing professionals in this study exhibited levels of perceived stress (PSS) classified as high. Similar results were found in studies with telephone nurses in Sweden^(25)^ and hospital nurses in Iran^(26)^. Higher levels of stress were observed in a study with PHC professionals in Brazil^(27)^, where average PSS scores were classified as very high. Conversely, lower averages were found in studies with newly hired nurses at medical centers in Taiwan^(28)^ and with hospital nurses in China^(29)^, both classified as having moderate PSS levels. All mentioned studies used the PSS.

PHC nursing professionals experience stress levels that, combined with working conditions, affect their mental health and increase the risk of developing other illnesses. A deeper understanding of this practice environment is needed to reduce stress by strengthening coping strategies^(30)^. High work demands, combined with a shortage of human resources and territorial overload, result in a workload that is the main stress-generating factor for PHC nursing professionals^(30,31)^. Nurses, in particular, also face role ambiguity, characterized by a lack of clarity about the tasks and responsibilities they must assume, as well as the need to perform activities that are not completed by other professionals, leading to an accumulation of tasks that extend beyond those expected in their role. These tasks are already numerous due to the nature of work in UBS^(31,32)^.

In addition to work overload, other stressors in PHC include inadequate infrastructure and environment, which hinder task execution and increase stress, as they fall beyond the professional’s control, limiting their ability to perform their work and generating feelings of anger and powerlessness, resulting in demotivation and dissatisfaction^(31,33)^. Lack of managerial support also impacts the stress levels of PHC nursing staff, as, in addition to low wages, limited promotion opportunities, and insufficient training, they face high productivity demands and lack autonomy to perform their work^(30,33-34)^. Moreover, close ties with the community may predispose workers to emotional distress due to feelings of powerlessness in dealing with local issues, as well as fears stemming from threats to their moral and physical integrity when working in open environments or in patients’ homes^(34)^.

Another important factor that may have contributed to the elevated stress levels in this study was the COVID-19 pandemic. Although data collection occurred at a time when the disease was more controlled in the country, studies conducted after the SARS (severe acute respiratory syndrome) outbreak in 2003 suggest that, beyond the immediate health impact, long-term effects were also observed among healthcare professionals^(14)^. Between one and two years after the SARS outbreak in Canada^(35)^, healthcare workers continued to exhibit signs of exhaustion, psychological distress, and post-traumatic stress disorder, with similar findings reported in Hong Kong one year after the SARS outbreak^(36)^.

In the present study, participants demonstrated high levels of SWBS and RWB and moderate levels of EWB. The SWBS and RWB results were higher than those found in studies conducted in Iran with hospital nurses^(37-40)^ and pre-hospital care providers^(13)^. For the EWB subscale, the results were similar to those in the aforementioned studies.

There was a difference between PSS levels and SWBS categories, with participants scoring higher on the SWBS scale showing lower PSS levels. This correlation between PSS and SWBS was also observed in studies conducted with nurses in the United States^(12,14)^, although it was classified as weak. Both religion and spirituality influence cognitive appraisal and negative emotional experiences related to stress, playing a significant role as coping strategies in stressful situations^(41)^. Spiritual well-being provides a sense of emotional support, which may enable individuals to reframe traumatic situations or disturbing events from a positive perspective^(10)^. The development and expression of spirituality help nursing teams cope with feelings of stress and burnout, resulting in a positive emotional response^(42)^. Workers with high SWBS scores experience reduced emotional fatigue. Spiritual health is associated with health-promoting behaviors, protection against the effects of stress, and a reduced risk of suicide^(8)^.

Regarding the subscales, RWB represents the vertical dimension of SWBS, or the connection with a superior being considered absolute^(10)^. Religion is a universal phenomenon that influences sociocultural norms and values, morality, and ideals, in addition to affecting individuals’ thinking and behavior^(41)^. Religious practices that may be used as stress-coping strategies include visiting places of worship, organizing spiritual activities, reading religious books, seeking refuge and protection in God, praying, and/or repeating sacred words or mantras^(41,43)^. In this study, despite the RWB score being classified as high, there was no significant correlation between RWB and PSS. Furthermore, no significant differences were observed between RWB categories and PSS levels among participants. A possible explanation for this finding relates to different religious coping styles. Stress researchers have identified three types of religious coping: self-direction (where the individual takes a more active role than God in solving problems), delegation (where the individual delegates to God the responsibility for solving their problems), and collaboration (where both the individual and God are active, sharing responsibility for problem resolution). Among these styles, only collaboration is positively associated with emotional adjustment for coping with stress^(41)^. Although religious coping styles were not measured in the present study, the use of styles not associated with positive outcomes may explain the lack of influence of RWB on PSS levels.

EWB corresponds to the horizontal dimension of SWBS, representing a personal interpretation of life that is independent of religious beliefs^(10)^. EWB is associated with practices of self-awareness and reflection, as well as the search for a deeper sense of life purpose^(41)^. Practices like mindfulness and other forms of meditation, such as compassion meditation, can help individuals find inner peace during difficult times, as the sense of belonging to something larger helps them understand they are not responsible for everything that happens in their lives and encourages acceptance of others without judgment^(41)^.

Another way spirituality can be used as a tool for coping with stress is through the discovery of life purpose. Purpose can be defined as a long-term intention directed toward achieving goals that are meaningful to the individual and have an impact on the external world^(44)^. A greater sense of life purpose is associated with microstructural features consistent with a healthier brain, including white matter and the right hippocampus^(45)^. Having a sense of purpose exerts a protective effect against the risk of psychopathologies, and individuals with a strong sense of life purpose tend to experience better psychophysical health throughout their lives^(44)^.

Work is a significant part of an individual’s life and affects their health in either a positive or negative way; work is never neutral^(46)^. It is associated with life purpose, which, in turn, is linked to vocation^(47)^. Nursing professionals may experience psychological distress and stress due to the nature of the profession, which involves emotionally intense interactions on a regular basis, along with working conditions that are not always ideal^(41)^. However, when work is aligned with an individual’s life purpose, it becomes meaningful^(47)^. The fact that a professional views their work as meaningful and in harmony with their life purpose can contribute to increased job satisfaction^(41)^.

Nursing professionals with a greater spiritual inclination tend to consider their profession more aligned with their psychological needs, leading them to see their work as a vocation. Spirituality also allows nurses to experience feelings of fulfillment, authenticity, and personal accomplishment, while strengthening their affection for the profession, increasing commitment to the organization, and reducing stress levels^(42)^.

Religion and spirituality are essential tools of emotional support that can help individuals rekindle their passion for both their profession and life^(43)^. It is essential for managers to recognize the importance of spiritual well-being for nursing professionals and to provide opportunities for discussions on the topic, as well as spaces for contemplation, reflection, and personal growth, along with religious and spiritual interventions. Such initiatives could help these workers reframe their work and find their life purpose, thereby protecting them against the effects of stress.

### Study limitations

Several limitations of this study should be noted. The measure of spiritual well-being used does not directly assess spirituality but rather the individual’s satisfaction with their life purpose and the deity they believe in, thus reflecting their relationship with their spirituality. This study did not measure religious coping styles or organizational and non-organizational religiosity. Variables that may directly impact stress levels, such as commuting distance, travel time, and the number of employment bonds, were also not evaluated. Additionally, it is important to highlight that in cross-sectional studies, as in this case, the relationship between the variables studied was assessed at a single point in time, making it impossible to establish a causal relationship between them. The results present limited external validity due to the lack of a sample size calculation. The studied group did not have a normal distribution, which required the use of non-parametric tests. Finally, data were collected solely from nursing professionals in PHC, which limits the generalizability of the results to populations in other settings.

### Contributions to nursing

The study suggests that spiritual well-being may play an important role in reducing high levels of stress among nursing professionals. Although there is substantial research on the influence of spirituality on patient health, few studies address spirituality and the health of nursing professionals. After an extensive literature review, no studies were found using the SWBS to assess the spirituality of nursing professionals in Brazil.

The results of this study indicate that spirituality may be an effective coping strategy for managing stressful situations arising from professional practice. To facilitate this, it is essential for health sector managers to create conditions that allow nursing staff to fully experience their spirituality. Factors such as adequate compensation, a 30-hour workweek, and rest infrastructure can provide individuals with the necessary time to dedicate to their quality of life, including religious and spiritual activities in their daily routine.

## CONCLUSIONS

In this study, participants with higher levels of spiritual well-being showed lower levels of perceived stress. There was a significant difference among low, moderate, and high categories of spiritual well-being and existential well-being with regard to perceived stress, with a medium effect size. Spiritual well-being and existential well-being were moderately negatively correlated with perceived stress levels. Simple regression analysis showed statistical significance for both spiritual well-being and existential well-being, suggesting that an increase in these variables could lead to a reduction in stress levels. Religious well-being was not correlated with perceived stress levels among participants in this study, nor was there a significant difference between its categories and the studied outcome.

Experimental studies are needed to verify the effectiveness of interventions aimed at increasing spiritual well-being in reducing stress among nursing professionals.

## Data Availability

*https://doi.org/10.48331/scielodata.TKCEZN*
