# Supplementary material for: Spiritual well-being and perceived stress in primary health care nursing professionals
Source: Rev Bras Enferm. 2025 Jun 20;78(2):e20240193. doi: 10.1590/0034-7167-2024-0193 (PMC12185147; doi:10.1590/0034-7167-2024-0193)
Supplement: Translation

# Translation: Bem-estar espiritual e estresse percebido em profissionais de enfermagem da atenção primária à saúde

**DOI:** 10.1590/0034-7167-2024-0193pt

**Authors:** Fabiana Gonçalves Seki Gava, Ruth Natalia Teresa Turrini

**Affiliations:** I Universidade de São Paulo. São Paulo, São Paulo, Brasil

**Keywords:** Estresse Psicológico, Espiritualidade, Enfermagem, Atenção Primária à, Saúde, Estudos Transversais.

## Abstract

**Objetivo:**

Verificar a relação entre o bem-estar espiritual e os níveis de estresse percebido em profissionais de enfermagem da atenção primária à saúde.

**Métodos:**

Estudo descritivo e correlacional realizado com 87 profissionais de enfermagem. Foram utilizados, para a coleta de dados, a Escala de Estresse Percebido e a Escala de Bem-estar Espiritual. Os dados foram submetidos à análise de correlação de Spearman, regressão linear simples e teste de Kruskal-Wallis.

**Resultados:**

Observou-se uma diferença significativa nos níveis de estresse percebido entre as categorias de bem-estar espiritual alto, moderado e baixo, com tamanho de efeito médio. O bem-estar espiritual apresentou uma correlação negativa moderada com os níveis de estresse percebido.

**Conclusão:**

Níveis mais altos de bem-estar espiritual estão associados a níveis mais baixos de estresse percebido, sugerindo que a espiritualidade pode ser uma ferramenta eficaz de enfrentamento ao estresse para profissionais de enfermagem da atenção primária.

## INTRODUÇÃO

O estresse percebido pode ser definido como uma situação que o indivíduo percebe como significativa, desafiadora ou ameaçadora, cujas demandas excedem seus recursos para enfrentá-la^(1)^. O estresse não deve ser compreendido como algo exclusivamente negativo a ser combatido. O corpo humano está adaptado para lidar com o estresse em pequenas doses, uma vez que ele estimula a ação, permitindo ao indivíduo concentrar sua atenção e tomar as melhores decisões frente às circunstâncias. É apenas quando a intensidade e a duração do estresse aumentam que ele se torna prejudicial. Altos níveis de estresse prolongado interferem no equilíbrio mental, nas habilidades cognitivas e funcionais, além de estarem associados a comportamentos prejudiciais à saúde, desenvolvimento de resistência à insulina, diabetes, síndrome metabólica, hipertensão arterial, osteoporose, obesidade central, dislipidemia e depressão^(2,3)^.

A atenção primária à saúde (APS) é a principal porta de entrada do usuário no sistema público de saúde e é responsável por resolver até 90% dos problemas de saúde da população^(4,5)^. A amplitude de sua atuação, somada às especificidades desse modelo de trabalho, pode levar ao desenvolvimento de problemas de saúde mental nos profissionais desse nível de atenção, especialmente entre os profissionais de enfermagem. O déficit de recursos humanos resulta em sobrecarga de trabalho e tempo insuficiente para a execução das atividades, o que, aliado a conflitos com a equipe e pacientes, ambiguidade de papéis, falta de suporte de superiores hierárquicos e impotência diante do sofrimento dos usuários, pode contribuir para o aumento dos níveis de estresse entre os trabalhadores de enfermagem da APS^(6)^. O estresse relacionado ao trabalho coloca esses profissionais em maior risco de desenvolver problemas de saúde física e mental, com impacto nas dimensões pessoal, familiar e profissional de suas vidas. Dessa forma, surge a necessidade de adotar estratégias de promoção da saúde e aumento da resiliência para essa população^(7,8)^, entre as quais se destaca o cultivo da espiritualidade.

A literatura não apresenta consenso sobre a definição de espiritualidade. No entanto, a maioria dos estudos a descreve como uma busca pessoal por significado e propósito de vida, por respostas às questões existenciais e pela compreensão da relação do indivíduo com o sagrado ou o transcendente, podendo ou não estar relacionada à religião^(9)^. A espiritualidade é uma dimensão presente em todos os seres humanos, enquanto a religião está ligada a crenças e práticas formais específicas. A espiritualidade pode ser vista como um elemento orientador, capaz de fornecer um senso de direção na busca pela melhoria da saúde física, socioemocional, intelectual, ocupacional e ambiental^(10)^.

O bem-estar espiritual é considerado uma dimensão da saúde e pode ser definido como um estado emocional positivo, com comportamentos construtivos e pensamentos edificantes nas relações consigo mesmo, com os outros e com uma dimensão além do tangível. Esse estado encoraja um sentimento de identidade, plenitude, contentamento, afeto, consideração, atitudes otimistas, serenidade interior, equilíbrio, propósito e sentido de vida^(10)^.

Quando o indivíduo vive segundo uma perspectiva fundamentada na espiritualidade, ele demonstra maior tolerância ao estresse físico e psicológico e manifesta melhor habilidade de enfrentamento diante de doenças graves e do isolamento, o que pode contribuir, inclusive, para um envelhecimento bem-sucedido^(11)^. A espiritualidade influencia positivamente a resiliência e a esperança, além de capacitar os profissionais de saúde a lidar com as experiências de dor, sofrimento e morte^(11)^. Ademais, o bem-estar espiritual está associado ao aumento da satisfação no trabalho e à melhoria da performance profissional^(12,13)^. Esse estado aprimora as habilidades de adaptação e a função mental e exerce um impacto benéfico no cuidado de enfermagem. Existe uma correlação positiva e significativa entre o cuidado de enfermagem e o bem-estar espiritual^(13)^.

Apesar do crescente número de pesquisas sobre a importância do cuidado espiritual, a maioria dos estudos concentra-se no cuidado espiritual destinado aos pacientes, e não aos profissionais de enfermagem. Poucos estudos investigam a relação entre o bem-estar espiritual e os níveis de estresse nessa população^(8)^. Dois estudos realizados nos Estados Unidos, um com enfermeiros pediátricos e outro com estudantes de enfermagem, indicaram uma correlação inversa e fraca entre os níveis de bem-estar espiritual e o estresse percebido^(12,14)^. Não foram encontrados estudos nacionais sobre essa temática.

## OBJETIVO

Verificar a relação entre o bem-estar espiritual e os níveis de estresse percebido em profissionais de enfermagem da APS.

## MÉTODOS

### Aspectos Éticos

O estudo foi conduzido em conformidade com as diretrizes éticas nacionais e internacionais, tendo sido aprovado pelos Comitês de Ética em Pesquisa da Escola de Enfermagem e da Faculdade de Saúde Pública da Universidade de São Paulo. O consentimento livre e esclarecido foi obtido de todos os participantes por meio eletrônico.

### Desenho, Período e Local do Estudo

Trata-se de um estudo descritivo e correlacional, elaborado de acordo com as recomendações do STROBE (Strengthening the Reporting of Observational Studies in Epidemiology) para estudos transversais^(15)^.

Os dados foram coletados em 32 das 40 Unidades Básicas de Saúde (UBS) do município de Osasco, SP, Brasil, e no Centro de Saúde Escola Geraldo de Paula Souza (CSEGPS), em São Paulo, SP, Brasil, no período de outubro de 2021 a junho de 2022.

### Amostra, Critérios de Inclusão e Exclusão

A população foi composta por auxiliares, técnicos de enfermagem e enfermeiros. A amostra não probabilística incluiu 87 profissionais de enfermagem atuantes nas UBS que participaram voluntariamente do estudo. Os critérios de inclusão foram identificar-se como estressado e estar trabalhando na instituição há pelo menos seis meses. Foram excluídos os profissionais que estavam de férias ou afastados de suas funções durante o período de coleta de dados.

### Protocolo de Estudo

Para os participantes das UBS de Osasco, o convite para participar da pesquisa foi enviado pelas coordenadoras de enfermagem do departamento de APS via mensagem de WhatsApp® no grupo de trabalho. Na mensagem, foram disponibilizados um vídeo explicativo e o link para o formulário eletrônico de intenção de participação, contendo informações de contato e perguntas referentes aos critérios de elegibilidade. No CSEGPS, o convite foi realizado durante uma reunião de equipe, na qual uma das pesquisadoras explicou os objetivos da pesquisa e distribuiu folhetos com um código QR direcionando para o formulário eletrônico de intenção de participação. Os participantes elegíveis receberam o formulário eletrônico de coleta de dados via WhatsApp®, contendo o Termo de Consentimento Livre e Esclarecido (TCLE), o instrumento biosociodemográfico, a Escala de Estresse Percebido (EP) e a Escala de Bem-estar Espiritual (BEEsp).

Ao clicar no link de acesso ao formulário de coleta de dados, os participantes foram automaticamente direcionados para a página do TCLE, que continha todas as informações sobre o estudo e o canal de comunicação com as pesquisadoras. Após a leitura do TCLE, o participante escolhia uma entre três opções: A) “Concordo em participar voluntariamente da pesquisa”; B) “Não concordo em participar da pesquisa”; C) “Tenho dúvidas sobre a pesquisa e gostaria de esclarecimentos antes de concordar com a minha participação”. O instrumento só pôde ser acessado caso o participante optasse pela opção A.

O instrumento biosociodemográfico incluía informações relacionadas à categoria profissional, sexo, estado civil, idade e número de filhos.

A EP mede o grau com que os indivíduos percebem as situações de sua vida como imprevisíveis, incontroláveis e sobrecarregadas^(16)^. O instrumento foi validado para o português do Brasil, apresentando consistência interna medida pelo alfa de Cronbach com valor de 0,82 no estudo de validação e 0,91 no presente estudo. A escala é composta por 14 questões em uma escala de Likert, cujas respostas variam de 0 (nunca) a 4 (sempre), sendo a pontuação invertida para as questões negativas. A soma das pontuações varia de zero a 56 pontos, podendo ser analisada de forma categórica em cinco intervalos de pontuação: < 18 = estresse baixo; 19-24 = estresse normal; 25-29 = estresse moderado; 30-35 = estresse alto; e > 35 = estresse muito alto^(17,18)^.

A BEEsp possui 20 itens divididos em duas subescalas, com dez questões cada: o Bem-estar Religioso (BER) mede a satisfação do indivíduo com Deus, enquanto o Bem-estar Existencial (BEEx) avalia se o indivíduo possui um propósito de vida, independentemente da religião^(19)^. A escala foi validada para o português brasileiro, apresentando consistência interna medida pelo alfa de Cronbach com valor de 0,92 para a escala geral, 0,92 para o BER e 0,85 para o BEEx^(20)^. No presente estudo, a consistência interna apresentou valores de 0,92 para a escala geral, 0,82 para o BER e 0,94 para o BEEx. As respostas são registradas em uma escala de Likert que varia de 1 (discordo totalmente) a 6 (concordo totalmente), com pontuação invertida para as questões negativas. A soma das pontuações varia de 20 a 120, e a categorização sugerida pelos autores é a seguinte: para o BEEsp, 20-40 = baixo bem-estar espiritual, 41-99 = moderado bem-estar espiritual, 100-120 = alto bem-estar espiritual; para a subescala BER, 20-40 = relação insatisfatória com Deus ou BER baixo, 21-49 = senso moderado de bem-estar religioso ou BER moderado, 50-60 = visão positiva do relacionamento com Deus ou BER alto; e para a subescala BEEx, 10-20 = baixa satisfação com a vida e possível falta de clareza sobre seu propósito de vida ou BEEx baixo, 21-49 = nível moderado de satisfação com a vida e seu propósito ou BEEx moderado, 50-60 = alto nível de satisfação com a vida e claro senso de propósito ou BEEx alto^(21)^.

### Análise dos Dados e Estatística

Para a caracterização da amostra, foram utilizadas medidas descritivas de frequências absolutas e relativas, bem como medidas de tendência central e variabilidade.

A correlação entre a EP e as escalas de BEEsp, BER e BEEx foi verificada por meio da correlação de Spearman. Para avaliar a magnitude das correlações, foram adotados os seguintes parâmetros: < 0,3 = correlação fraca, 0,3 a < 0,7 = correlação moderada e ≥ 0,7 = correlação forte. As variáveis independentes que apresentaram correlação significativa com o desfecho foram incluídas em um modelo de regressão linear simples.

Para comparar os níveis de EP de acordo com as categorias de BEEsp e suas subescalas, foi utilizado o teste de Kruskal-Wallis, seguido do teste post-hoc de Dunn para comparação entre categorias. O tamanho de efeito foi medido pelo eta quadrado de postos (rank eta squared - η^2^), com as seguintes classificações: 0,02 = pequeno, 0,13 = médio e 0,26 = grande^(22)^.

Foram realizadas análises de regressão linear simples bivariadas entre as variáveis sociodemográficas e o BEEsp, sendo que aquelas com p < 0,2 foram incluídas em um modelo de regressão linear múltipla juntamente com o BEEsp.

Os dados foram armazenados em uma planilha do Microsoft Excel® e analisados no software JAMOVI, versão 2.4.2. O nível de significância adotado foi de 5%.

## RESULTADOS

No total 113 profissionais de enfermagem preencheram o formulário eletrônico de intenção de participação. Houve uma perda de 26 participantes, dois por não atenderam aos critérios de inclusão e 24 desistiram de participar do estudo antes do preenchimento do instrumento de coleta de dados, o que resultou em uma amostra de 87 participantes.

A Tabela 1 apresenta as características biosociodemográficas dos participantes do estudo. A mediana de EP é compatível a classificação de estresse alto, com uma prevalência de 52,8% (n=46) dos participantes com níveis de estresse alto e muito alto. A mediana de BEEsp classifica os participantes na faixa do bem-estar espiritual alto (50,6%, n=44). Com relação às subescalas, suas medianas classificam os participantes como tendo uma visão positiva do relacionamento com Deus (BER alto - 64,4%, n=56), e como moderado nível de satisfação com a vida e senso de propósito (BEEx moderado - 67,8%, n=59).

**Tabela 1:** Características biosociodemográficas dos participantes. Osasco e São Paulo, São Paulo, Brasil, 2022

Variáveis qualitativas (N=87)	n (%)	
Sexo		
Feminino	80 (92,0)	
Masculino	7 (8,0)	
Estado civil		
Solteiro	20 (23,0)	
Casado ou em união estável	50 (57,5)	
Divorciado	14 (16,1)	
Viúvo	3 (3,4)	
Categoria profissional		
Nível técnico	61 (70,1)	
Enfermeiro	26 (29,9)	
**Variáveis quantitativas (N=87)**	**Média (DP)**	**Mediana (1ºQ; 3ºQ)**
Idade	44,7 (9,3)	44,0 (38,0; 50,5)
Número de filhos	1,6 (1,2)	1,0 (1,0; 2,0)
EP	31,1 (8,2)	30,0 (25,5; 37,5)
BER	50,1 (12,3)	55,0 (43,0; 60,0)
BEEx	42,2 (9,6)	43,0 (36,5; 50,0)
BEEsp	92,2 (20,0)	100,0 (82,5; 106,0)

*Nota: BEEx = bem-estar existencial. BEEsp = bem-estar espiritual. BER = bem-estar religioso. DP = desvio-padrão. EP = estresse percebido. 1ºQ = primeiro quartil. 3ºQ = terceiro quartil.*

Ao observar a distribuição dos escores de EP de acordo com as categorias de BEEsp e suas subescalas, verifica-se que, quanto mais alto o bem-estar espiritual, menores os níveis de EP (Figura 1). Nos participantes com BEEsp, BER e BEEx altos, o valor da mediana de EP foi classificado como moderado. Já nos participantes com BEEsp, BER e BEEx moderados, o valor da mediana de EP foi classificado como alto. Por último, nos participantes com BEEsp, BER e BEEx baixos, o valor da mediana de EP foi classificado como muito alto.

**Figura 1:** Distribuição da mediana dos escores de EP nas categorias de bem-estar espiritual e suas subescalas. Osasco e São Paulo, São Paulo, Brasil, 2022

*Nota: BEEsp = bem-estar espiritual. BER - bem-estar religioso. BEEx = bem-estar existencial. EP = estresse percebido.*

Foi realizado o teste de Kruskal-Wallis para a comparação dos valores da mediana de EP entre as categorias do BEEsp, BEEx e BER. Houve diferença significativa entre as categorias para o BEEsp (p = 0,002) e BEEx (p = <0,001), mas não para o BER (p = 0,06). Na sequência, realizou-se o teste *post-hoc* de comparações múltiplas de Dunn para verificar a diferença de EP entre as categorias de bem-estar espiritual, para as variáveis com diferença estatisticamente significativas. Para o BEEsp, o teste mostrou diferenças significativas entre as categorias alto e moderado (p = 0,015), alto e baixo (p = 0,004), e moderado e baixo (p = 0,05), com tamanho de efeito médio (η^2^ = 0,119). O mesmo aconteceu para o BEEx, que apresentou diferenças entre as categorias alto e moderado (p = 0,004), alto e baixo (p<0,001), e moderado e baixo (p = 0,011), também com tamanho de efeito médio (η^2^ = 0,180).

Ao analisar a associação entre o bem-estar espiritual e o estresse, observou-se que o BEEsp e o BEEx apresentaram correlação negativa moderada e significativa com os níveis de EP dos participantes. O BER não apresentou correlação significativa com o EP, conforme visto na Tabela 2.

**Tabela 2:** Correlação entre EP, BEEsp e suas subescalas. Osasco e São Paulo, São Paulo, Brasil, 2022

Variáveis	EP	BEEsp	BEEx	BER
EP	1,000	--	--	--
BEEsp	-0,367^*^	1,000	--	--
BEEx	-0,519^*^	0,897^*^	1,000	--
BER	-0,122	0,842^*^	0,568^*^	1,000

Nota:

* p < 0,001. BEEsp = bem-estar espiritual. BEEx = bem-estar existencial. BER = bem-estar religioso. EP = estresse percebido.

O BEEsp e o BEEx foram inseridos em uma análise de regressão linear simples para verificar sua relação com a variável EP. O BER não foi inserido por não apresentar correlação significativa com a variável de desfecho. Neste modelo, tanto o BEEx (p<0,001; r^2^ = 0,304) quanto o BEEsp (p<0,001; r^2^ = 0,154) mostraram-se negativamente associados com o EP.

As variáveis sociodemográficas não se relacionaram com os níveis de EP: idade (p = 0,697), número de filhos (p = 0,959), estado civil (p = 0,146) e categoria profissional (p = 0,405). O estado civil, por apresentar p < 0,2 foi inserido em um modelo de regressão múltipla juntamente com o BEEsp, mas a análise não mostrou resultados significativos nas categorias do estado civil (casado-solteiro, p = 0,361; divorciados-solteiro, p = 0,509; viúvo-solteiro, p = 0,714). Neste modelo, somente o BEEsp mostrou associação com os níveis de EP (p = 0,001).

## DISCUSSÃO

A maioria dos participantes do estudo era composta por mulheres, profissionais de nível médio, que viviam com um companheiro, com idade média superior a 40 anos e poucos filhos. A enfermagem é uma profissão historicamente exercida por mulheres. Embora o número de homens na enfermagem tenha aumentado nos últimos 30 anos, eles ainda representam apenas 14,1% dos trabalhadores da área^(23)^. A maior parte dos participantes estava na terceira fase da vida profissional, a fase da maturidade, que inclui indivíduos entre 36 e 50 anos^(24)^. Esses resultados são semelhantes aos de um estudo realizado com trabalhadores da enfermagem no Brasil durante a pandemia de COVID-19^(23)^, mas diferem do perfil geral da enfermagem no país, onde 61,7% dos profissionais têm idade inferior a 40 anos^(24)^. Os dados referentes à categoria profissional e ao estado civil também estão em consonância com o perfil da enfermagem no Brasil, onde 77% dos profissionais possuem nível técnico e 48,7% são casados, estão em união estável ou em contrato consensual^(24)^.

Os profissionais de enfermagem que participaram do estudo apresentaram níveis de EP classificados como altos. Resultados semelhantes foram encontrados em estudos com enfermeiros telefonistas na Suécia^(25)^ e enfermeiros hospitalares no Irã^(26)^. Níveis de estresse mais elevados foram observados em um estudo com profissionais da APS no Brasil^(27)^, em que as médias de EP foram classificadas como muito altas. Em contrapartida, médias inferiores foram encontradas em estudos com enfermeiros recém-contratados em centros médicos em Taiwan^(28)^ e com enfermeiros atuantes em hospitais na China^(29)^, ambos com EP classificado como moderado. Todos os estudos mencionados utilizaram a EP.

Os profissionais de enfermagem da APS experimentam níveis de estresse que, associados às condições de trabalho, afetam sua saúde mental e aumentam o risco de desenvolver outras doenças. É necessário aprofundar o conhecimento sobre esse cenário de prática, visando reduzir o estresse por meio do fortalecimento das estratégias de enfrentamento^(30)^. As altas demandas de trabalho, somadas ao déficit de recursos humanos e à sobrecarga territorial, resultam em uma carga de trabalho que representa o principal fator gerador de estresse na enfermagem da APS^(30,31)^. Os enfermeiros, em particular, também enfrentam a ambiguidade de papéis, caracterizada pela falta de clareza sobre as tarefas e responsabilidades que devem assumir, bem como pela necessidade de realizar atividades que não são executadas por outros profissionais, acumulando atribuições que vão além das previstas para seu núcleo profissional. Essas atribuições já são numerosas devido à própria natureza do trabalho nas UBS^(31,32)^.

Além da sobrecarga de trabalho, outros elementos estressores na APS incluem a precariedade da estrutura e do ambiente, que dificultam a realização das atividades, aumentando o estresse, pois fogem da capacidade de resolução do profissional, limitam a execução de seu trabalho e geram sentimentos de raiva e impotência, resultando em desmotivação e insatisfação^(31,33)^. A falta de apoio da gestão também impacta o estresse dos trabalhadores de enfermagem na APS, uma vez que, além dos baixos salários, da falta de oportunidades de promoção e de capacitação, eles enfrentam a exigência de altos níveis de produtividade e falta de autonomia para realizar seu próprio trabalho^(30,33,34)^. Além disso, o vínculo estreito com o território pode predispor o trabalhador ao sofrimento, devido à sensação de impotência diante dos problemas a serem tratados, além do medo gerado por ameaças à integridade moral e física do profissional que atua em ambientes abertos ou nas residências dos usuários^(34)^.

Outro fator importante que pode ter contribuído para os níveis elevados de estresse no presente estudo foi a pandemia de COVID-19. Embora a coleta de dados tenha ocorrido em um momento de maior controle da doença no país, estudos realizados após o surto de SARS (síndrome respiratória aguda grave) em 2003 sugerem que, além do impacto imediato sobre a saúde, também foram observados efeitos sustentados a longo prazo entre os profissionais de saúde^(14)^. Entre um e dois anos após o surto de SARS no Canadá^(35)^, os trabalhadores da saúde ainda apresentavam esgotamento, sofrimento psicológico e transtorno de estresse pós-traumático, com resultados semelhantes sendo encontrados em Hong Kong, um ano após o surto de SARS^(36)^.

No presente estudo, os participantes apresentaram níveis altos de BEEsp e BER, e níveis moderados de BEEx. Os resultados de BEEsp e BER foram superiores aos encontrados em pesquisas realizadas no Irã com enfermeiros hospitalares^(37-40)^ e de atendimento pré-hospitalar^(13)^. Para a subescala BEEx, os resultados encontrados foram semelhantes aos dos estudos citados.

Houve diferença entre os níveis de EP e as categorias de BEEsp, com os participantes que obtiveram maior pontuação na escala de BEEsp apresentando menores níveis de EP. Essa correlação entre EP e BEEsp também foi observada em estudos realizados com enfermeiros nos Estados Unidos^(12,14)^, embora classificada como fraca. Tanto a religião quanto a espiritualidade regulam a avaliação cognitiva e as experiências emocionais negativas associadas ao estresse, desempenhando um papel significativo como estratégia de enfrentamento em situações estressantes^(41)^. O bem-estar espiritual proporciona uma sensação de suporte emocional, o que pode levar o indivíduo a ressignificar situações traumáticas ou eventos perturbadores a partir de uma perspectiva positiva^(10)^. O desenvolvimento e a expressão da espiritualidade ajudam a equipe de enfermagem a lidar com sentimentos de estresse e esgotamento, resultando em uma reação emocional positiva^(42)^. Trabalhadores com elevada pontuação de BEEsp apresentam redução da fadiga emocional. A saúde espiritual está associada a comportamentos de promoção à saúde, protege contra os efeitos do estresse e diminui o risco de suicídio^(8)^.

Com relação às subescalas, o BER corresponde ao sentido vertical do BEEsp, ou seja, à ligação com um ser superior considerado absoluto^(10)^. A religião é um fenômeno universal que influencia normas e valores socioculturais, moralidade e ideais, além de impactar o processo de pensamento e comportamento dos indivíduos^(41)^. As práticas religiosas que podem ser utilizadas como estratégia de enfrentamento ao estresse incluem visitar locais de adoração, organizar atividades espirituais, ler livros religiosos, buscar abrigo e proteção em Deus, orar e/ou repetir palavras sagradas ou mantras^(41,43)^. Neste estudo, apesar da pontuação do BER ser classificada como alta, não houve correlação significativa entre o BER e o EP. Além disso, não se observou diferença significativa entre as categorias de BER e os níveis de EP dos participantes. Uma possível explicação para essa ocorrência relaciona-se aos estilos de coping religioso. Pesquisadores do estresse identificaram três tipos de coping religioso: autodireção (o indivíduo é mais ativo que Deus na resolução dos problemas), delegação (o indivíduo delega a Deus a responsabilidade por resolver seus problemas) e colaboração (tanto o indivíduo quanto Deus são ativos, compartilhando a responsabilidade para a resolução dos problemas). Desses estilos, apenas a colaboração está positivamente associada ao ajuste emocional para o enfrentamento do estresse^(41)^. Embora os estilos de coping religioso não tenham sido medidos no presente estudo, o uso de estilos não associados a desfechos positivos pode ser uma explicação para a ausência de influência do BER nos níveis de EP.

O BEEx corresponde à dimensão horizontal do BEEsp, uma interpretação pessoal da vida que independe de crenças religiosas^(10)^. O BEEx está associado a práticas de autoconhecimento e reflexão e à busca de um sentido de vida mais profundo^(41)^. Assim, práticas como o mindfulness e outros tipos de meditação, como a meditação da compaixão, podem ajudar o indivíduo a encontrar paz interior durante momentos difíceis, pois o senso de pertencer a algo maior ajuda a compreender que ele não é responsável por tudo o que acontece em sua vida e a aceitar os outros sem julgamento^(41)^.

Outro mecanismo pelo qual a espiritualidade pode ser usada como ferramenta de enfrentamento ao estresse é a descoberta de um propósito de vida. O propósito pode ser definido como uma intenção de longo prazo voltada para a realização de objetivos significativos para o indivíduo e que causem impacto no mundo externo^(44)^. Um maior senso de propósito de vida está associado a características microestruturais consistentes com as de um cérebro mais saudável, incluindo a substância branca e o hipocampo direito^(45)^. Ter um senso de propósito exerce um efeito protetor contra o risco de psicopatologias, e indivíduos com um forte propósito de vida tendem a apresentar melhor saúde psicofísica ao longo da vida^(44)^.

O trabalho é uma parte significativa na vida dos indivíduos e afeta sua saúde, de maneira positiva ou negativa. O trabalho nunca é neutro^(46)^. Ele está associado ao propósito de vida, que, por sua vez, está ligado à vocação^(47)^. Os profissionais de enfermagem podem ser acometidos pelo sofrimento psicológico e pelo estresse devido à natureza da profissão, que envolve interações emocionalmente intensas de forma regular, além de condições de trabalho que nem sempre são ideais^(41)^. No entanto, quando o trabalho está alinhado com o propósito de vida do indivíduo, ele se torna significativo^(47)^. O fato de o profissional considerar seu trabalho significativo e em sintonia com seus propósitos de vida pode contribuir para o aumento da satisfação no trabalho^(41)^.

Os trabalhadores da enfermagem com maior inclinação espiritual tendem a considerar sua profissão mais congruente com suas necessidades psicológicas, levando-os a ver seu trabalho como uma vocação. A espiritualidade também permite que enfermeiros experienciem sentimentos de plenitude, autenticidade e realização pessoal, além de intensificar o afeto pela profissão, aumentando o compromisso com a organização e reduzindo os níveis de estresse^(42)^.

A religião e a espiritualidade são ferramentas essenciais de suporte emocional que podem ajudar os indivíduos a reencontrarem a paixão tanto pela profissão quanto pela vida^(43)^. É necessário que os gestores se conscientizem da importância do bem-estar espiritual para os profissionais de enfermagem e providenciem momentos para o debate sobre o tema, espaços para contemplação, reflexão e crescimento pessoal, além de intervenções de natureza religiosa e espiritual. Tais iniciativas permitiriam a esses trabalhadores ressignificar o trabalho e encontrar seu propósito de vida, protegendo-os contra os efeitos do estresse.

### Limitações do Estudo

Algumas limitações do presente estudo devem ser destacadas. O bem-estar espiritual avaliado não mede diretamente a espiritualidade, mas sim a satisfação do indivíduo com seu propósito de vida e com a divindade na qual acredita, refletindo, assim, sua relação com a própria espiritualidade. Neste estudo, não foram mensurados os estilos de coping religioso nem a religiosidade organizacional e não organizacional. Variáveis que podem impactar diretamente os níveis de estresse, como distância até o trabalho, tempo de deslocamento e número de vínculos empregatícios, também não foram avaliadas. Além disso, é importante ressaltar que, em pesquisas de abordagem transversal, como é o caso deste estudo, a conexão entre as variáveis estudadas foi avaliada em um único momento, o que impossibilita o estabelecimento de uma relação causal entre elas. Os resultados apresentam validade externa limitada devido à ausência de cálculo amostral. O grupo estudado não apresentou distribuição normal, o que exigiu o uso de testes não paramétricos. Por fim, os dados foram coletados apenas entre profissionais de enfermagem da APS, o que impede a generalização dos resultados para populações em outros contextos.

### Contribuições para a Área da Enfermagem

O estudo sugere que o bem-estar espiritual pode desempenhar um papel importante na redução dos elevados níveis de estresse entre os profissionais de enfermagem. Há muitas pesquisas sobre a influência da espiritualidade na saúde dos pacientes, mas poucos estudos abordam a espiritualidade e a saúde dos profissionais de enfermagem. Após extensas buscas na literatura, não foram encontrados estudos que utilizassem a BEEsp para avaliar a espiritualidade de profissionais de enfermagem no Brasil.

Os resultados deste estudo indicam que a espiritualidade pode ser uma estratégia de enfrentamento eficiente para lidar com as situações estressantes decorrentes da prática profissional. Para isso, é fundamental que gestores na área da saúde promovam condições que permitam aos trabalhadores de enfermagem experienciar sua espiritualidade de forma plena. Fatores como remuneração adequada, jornada semanal de 30 horas e infraestrutura para descanso podem proporcionar ao indivíduo o tempo necessário para dedicar-se à sua qualidade de vida, incluindo atividades religiosas e espirituais em seu cotidiano.

## CONCLUSÕES

No presente estudo, os participantes com maiores níveis de bem-estar espiritual apresentaram menores níveis de estresse percebido. Houve diferença significativa entre as categorias baixo, moderado e alto das variáveis bem-estar espiritual e bem-estar existencial em relação ao estresse percebido, com tamanho de efeito médio. O bem-estar espiritual e o bem-estar existencial apresentaram correlação negativa moderada com os níveis de estresse percebido. A análise de regressão simples mostrou significância estatística para o bem-estar espiritual e o bem-estar existencial, sugerindo que um aumento nos níveis dessas variáveis pode levar a uma redução nos níveis de estresse. O bem-estar religioso não apresentou correlação com os níveis de estresse percebido dos participantes neste estudo, assim como não houve diferença significativa entre suas categorias e o desfecho estudado.

Estudos com delineamento experimental são necessários para verificar a eficácia de intervenções que aumentem o bem-estar espiritual na redução do estresse dos profissionais de enfermagem.

## Data Availability

https://doi.org/10.48331/scielodata.TKCEZN
